# A reduction in Ptprq associated with specific features of the deafness phenotype of the miR-96 mutant mouse diminuendo

**DOI:** 10.1111/ejn.12484

**Published:** 2014-01-22

**Authors:** Jing Chen, Stuart L Johnson, Morag A Lewis, Jennifer M Hilton, Andreea Huma, Walter Marcotti, Karen P Steel

**Affiliations:** 1 Wellcome Trust Sanger InstituteCambridge, UK; 2 Wolfson Centre for Age-Related Diseases, King's College LondonGuy's Campus, London, SE1 1UL, UK; 3 Department of Biomedical Science, University of SheffieldSheffield, UK

**Keywords:** ear development, hereditary hearing loss, knockout and transgenic m, molecular genetics, sensory hair cells

## Abstract

miR-96 is a microRNA, a non-coding RNA gene which regulates a wide array of downstream genes. The miR-96 mouse mutant diminuendo exhibits deafness and arrested hair cell functional and morphological differentiation. We have previously shown that several genes are markedly downregulated in the diminuendo organ of Corti; one of these is *Ptprq*, a gene known to be important for maturation and maintenance of hair cells. In order to study the contribution that downregulation of *Ptprq* makes to the diminuendo phenotype, we carried out microarrays, scanning electron microscopy and single hair cell electrophysiology to compare diminuendo mutants (heterozygous and homozygous) with mice homozygous for a functional null allele of *Ptprq*. In terms of both morphology and electrophysiology, the auditory phenotype of mice lacking Ptprq resembles that of diminuendo heterozygotes, while diminuendo homozygotes are more severely affected. A comparison of transcriptomes indicates there is a broad similarity between diminuendo homozygotes and Ptprq-null mice. The reduction in Ptprq observed in diminuendo mice appears to be a major contributor to the morphological, transcriptional and electrophysiological phenotype, but does not account for the complete diminuendo phenotype.

## Introduction

The diminuendo mutant mouse exhibits deafness and arrested development of the cochlear sensory hair cells. Inner and outer hair cells (IHCs and OHCs) in homozygotes appear immature at young stages and later degenerate; by postnatal day (P)28, very few OHCs are visible. Heterozygotes display poor hearing at P15 followed by rapid deterioration until they lack any response to sound. Their hair cells also appear immature, but are still present at P28 (Lewis *et al*., 2009; Kuhn *et al*., 2011). In humans, two *MIR96* mutations have been found to be associated with dominantly-inherited progressive hearing loss (Mencia *et al*., 2009).

The diminuendo causative mutation is a single nucleotide change in the seed region of the microRNA miR-96, which disrupts its function by both loss of its proper targets and gain of novel ones (Lewis *et al*., 2009). miR-96 is a regulator controlling a wide array of genes, as seen in the microarrays comparing wildtypes and homozygotes (Lewis *et al*., 2009). However, a few genes were markedly downregulated in diminuendo homozygotes, including *Ptprq*, which is downregulated to ∼ 50% of the wildtype level (Lewis *et al*., 2009).

*Ptprq* is a phosphatidylinositol phosphatase, which in the mouse is expressed specifically in the hair bundles of cochlear and vestibular hair cells at early postnatal stages, although expression in the OHCs is transient. In mature hair cells, the staining is restricted to the base of the hair bundle (Goodyear *et al*., 2003). Three isoforms have been identified in chick hair cells, one expressed at immature stages and two in mature hair cells (Nayak *et al*., 2011). Ptprq localisation at the base of the stereocilia is actively maintained by Myo6, and is thought to be important for hair bundle maturation and maintenance (Goodyear *et al*., 2003; Sakaguchi *et al*., 2008). Mice homozygous for a catalytically inactive form of Ptprq (Ptprq-CAT-KO), which has a shortened intracellular domain but a wildtype C-terminus, do not respond to sound. It is likely to be a functional null; no Ptprq protein is detectable in the hair cells of homozygous Ptprq–CAT-KO mice (Goodyear *et al*., 2003). At early postnatal stages hair cells are present but the hair bundles are disorganised, and smaller and rounder in shape. At 3 months old hair cells are absent from the basal turn of the organ of Corti. The shaft connectors of vestibular hair cells are absent from Ptprq-CAT-KO homozygotes, as are vestibular evoked potentials (Goodyear *et al*., 2012).

miR-96 is a master regulator, controlling multiple genes and coordinating maturation of the hair cells (Kuhn *et al*., 2011), and its absence would be expected to affect a wide range of genes, although the pathways it regulates are not yet known. Understanding how much the downregulation of *Ptprq* contributes to the diminuendo phenotype is important for understanding the overall action of miR-96. We therefore undertook a detailed morphological examination of the hair cells, electrophysiology and transcriptome of diminuendo homozygotes and heterozygotes, Ptprq-CAT-KO homozygotes and controls, to ascertain what contribution the downregulation of *Ptprq* makes to the diminuendo phenotype.

## Materials and methods

### Mice

Mouse studies were carried out in accordance with UK Home Office regulations and the UK Animals (Scientific Procedures) Act of 1986 (ASPA) under a UK Home Office licence, and the study was approved by the Wellcome Trust Sanger Institute's Ethical Review Committee. Mice were culled using methods approved under this licence to minimize any possibility of suffering.

Two genetically modified mouse lines were used; the diminuendo line and the Ptprq-CAT-KO line. The diminuendo phenotype is the result of an *N*-ethyl-*N*-nitrosourea (ENU) mutagenesis screen (Lewis *et al*., 2009); the line was generated and has been maintained on a C3HeB/FeJ background. The Ptprq-CAT-KO allele is a targeted knock-out in which a 1061 bp fragment of Ptprq, which contains two of the exons coding for the catalytic domain, is replaced by a neomycin resistance cassette. 129/Sv stem cells were used, and the mouse line was established and has been maintained on a mixed C57BL/6J/129/Sv background (Goodyear *et al*., 2003). Fifty-eight animals were used in this study in total.

### Scanning electron microscopy

**Fig. 1:**
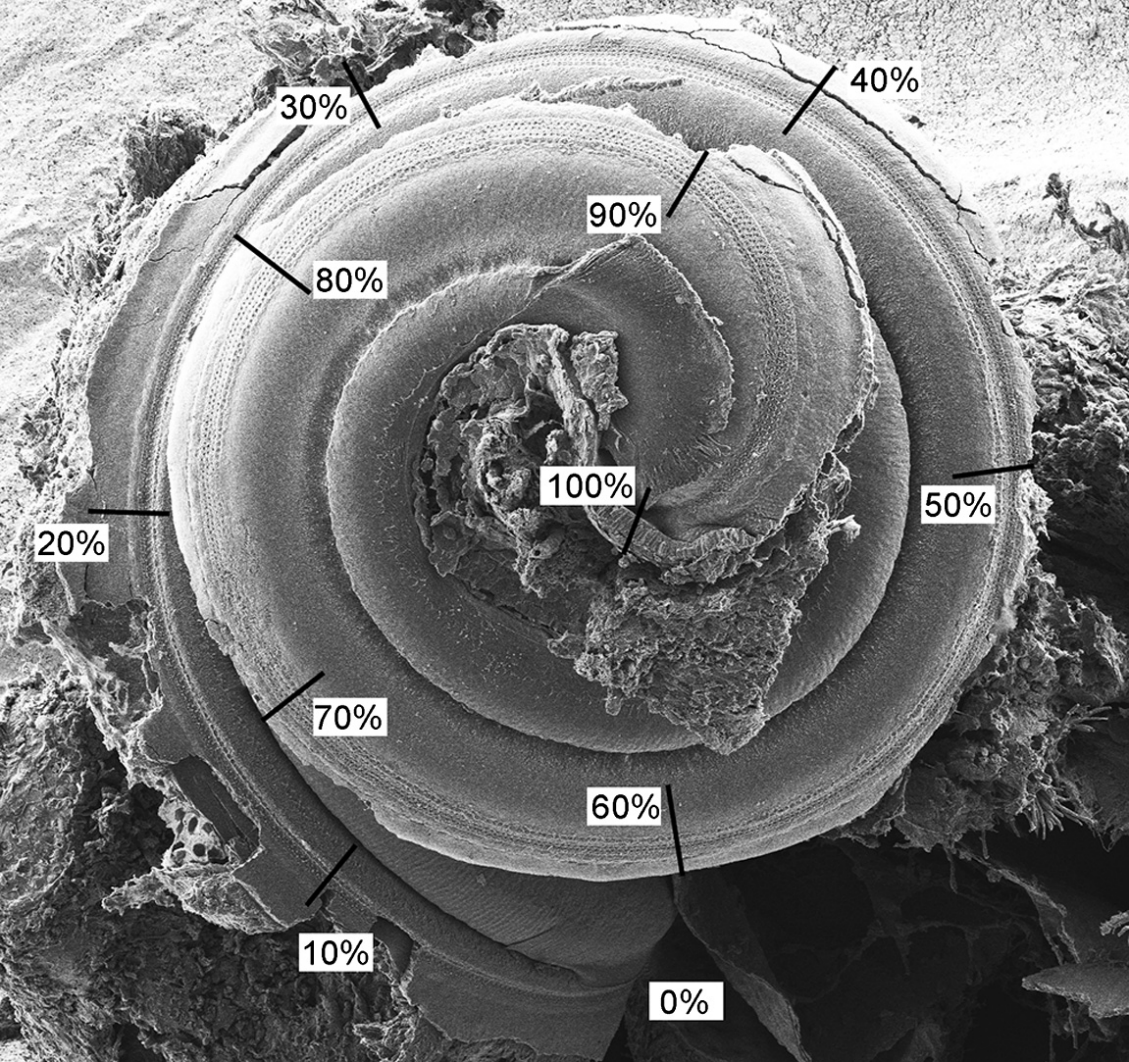
Method of measuring the length of the cochlear duct to make comparisons in corresponding positions. In order to compare hair cell bundles in corresponding positions along cochlear ducts between wildtype and mutant mice or between different mutant mice, the images were taken under a standard magnification (× 80) to show the whole organ of Corti. The cochlear duct was divided into ten parts of 10% from base to apex. A higher magnification (× 15 k) was used for analysis of IHCs and the outermost row of OHCs at every 10% interval along the length of the cochlear duct. Scale bar, 300 μm.

Scanning electron microscopy was carried out on diminuendo homozygote, heterozygote and wildtype and Ptprq-CAT-KO homozygote and wildtype mouse inner ears at P4 (four mice of each genotype, of either sex) as described in Chen *et al*. (2013), using the OTOTO method (Hunter-Duvar, 1978). Images were taken using an Hitachi SE-4800 microscope at 5 kV under a standard magnification (× 80) to show the whole organ of Corti and a higher magnification (× 15 k) for IHCs and the outermost row of OHCs at 10% intervals along the length of the cochlear duct from 10 to 90% of the distance from the base to apex (Fig. 1). No obvious differences of the size of the organ of Corti were observed during dissection of the different genotypes, and all mice examined had the normal number of cochlear turns. Cartoons of hair cells typical of each point along the organ of Corti and genotype were drawn based on the micrographs, to summarise their appearance from a similar angle.

### Microarray

Organ of Corti dissection and RNA extraction were carried out as described in Lewis *et al*. (2009), using male Ptprq-CAT-KO homozygous (*n* = 6) and wildtype (*n* = 6) P4 littermates collected within a 2-h time window to control for circadian changes. Total RNA (500 ng) for each sample was amplified and purified using the Illumina TotalPrep-96 RNA Amplification kit (Ambion, UK), according to the manufacturer's instructions. Biotin-labelled cRNA was then normalized to a concentration of 150 ng/uL and 1500 ng was hybridised to Illumina MouseWG-6 v2 beadarrays (Illumina, CA, USA) for 16 h (overnight) at 58 °C. Following hybridisation, beadarrays were washed and stained with streptavidin-cy3 (GE Healthcare, UK). Beadarrays were then scanned using the Beadarray reader and image data was then processed using Genome Studio software (Illumina). The data were normalised using a quantile normalisation, making the assumption that the overall intensity distributions of the arrays should be comparable (Bolstad, 2001; Bolstad *et al*., 2003). Normalised data was then analysed using the Bioconductor LUMI and LIMMA packages (Gentleman *et al*., 2004; Smyth, 2004; Smyth *et al*., 2005). The *P*-value was adjusted for multiple tests as described in Benjamini & Hochberg (1995), and the *P*-value cut-off was set to 0.05. Probe data from wildtype mice was compared to probe data from homozygote mutant mice; the only difference between each pair of littermates was the genotype. Microarray data are available in the ArrayExpress database (http://www.ebi.ac.uk/arrayexpress) under accession number E-MTAB-1785. Further analysis was carried out using the R statistical software package (Team, 2012) and the GSEA (Gene Set Enrichment Analysis) software (Mootha *et al*., 2003; Subramanian *et al*., 2005) on ranked microarray gene lists, using gene pathway sets downloaded from mSigDB (http://www.broadinstitute.org/gsea/msigdb/index.jsp, August 2013), Reactome (http://www.reactome.org/, July 2013) and Pathway Commons (http://www.pathwaycommons.org, July 2013). GSEA does not accept duplicate gene IDs, so if duplicates were present in the input gene list the highest absolute value was used.

### cDNA creation and RTPCR

Each pair of RNA samples (homozygote and wildtype Ptprq-CAT-KO P4 littermates of either sex; fourteen mice used in total) were normalised to the same concentration before treatment with DNAse 1 (Sigma; cat.no. AMP-D1). cDNA was created using the Superscript II Reverse Transcriptase kit (Invitrogen; cat. no. 11904-018). Primer/probe sets and the Taqman Master Mix were purchased from Applied Biosystems (Master Mix: 4364340; *Hprt1*: Mm01318747_g1; *Jag1*: Mm01270190_m1; *Calb2*: Mm00801461_m1; *Grxcr1*: Mm01217856_m1; *Hsd17b7*: Mm00501703_m1. Primer/probe sets for *Chrna1, Chrna9* and *Otof* were manually designed using a tool supplied by Applied Biosystems). *Hprt1* was used as the internal control, and *Jag1*, which is expressed in supporting cells (Morrison *et al*., 1999; Zine *et al*., 2000; Lewis *et al*., 2009), was used to control for sensory tissue content. Pairs were only used if their *Jag1* expression levels did not differ significantly (where significance was determined to be *P* < 0.05). Quantitative PCR was run on an ABI7000 machine (Applied Biosystems). Four technical replicates were carried out per sample, and three wildtype–homozygote pairs were tested for each probe. The 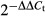 test was used to determine relative expression levels for each probe (Livak & Schmittgen, 2001); the calibrator was the wildtype littermate threshold for the same probe and the internal control was *Hprt*. Student's *t*-test was used for results which were both parametric and homoscedastic, Welch's *t*-test for parametric, heteroscedastic data, the Wilcoxon test for non-parametric, homoscedastic data and the Brunner-Munzel test for results which were neither parametric nor homoscedastic. All significance tests were non-directional, and a significance level of α < 0.05 was used. The *F*-test and Fligner–Killeen test were used to estimate homoscedasticity, and the Shapiro–Wilks test for normality. The Bonferroni test was used to adjust *P*-values to take multiple testing into account. Excel (Microsoft, Seattle, USA) and R (Team, 2012) were used to carry out the calculations and statistical tests.

### Single hair cell electrophysiology

Outer hair cells from the apical coil of diminuendo homozygote, heterozygote and wildtype mice of either sex were studied in acutely dissected organs of Corti from P1 to P6, where the day of birth is P0. Twenty-four mice were used in total. Animals were killed by cervical dislocation in accordance with UK Home Office regulations. Cochleae were dissected as previously described (Johnson *et al*., 2012) in normal extracellular solution (in mm): NaCl, 135; KCl, 5.8; CaCl_2_, 1.3; MgCl_2_, 0.9; NaH_2_PO_4_, 0.7; d-glucose, 5.6; and Hepes–NaOH, 10. Sodium pyruvate (2 mm), MEM amino acids solution (50 ×, without l-glutamine) and MEM vitamins solution (100 ×) were added from concentrates (Fisher Scientific, UK). The pH was adjusted to 7.5 (osmolality ∼ 308 mmol/kg). All animals were genotyped as previously described (Lewis *et al*., 2009).

Voltage recordings were performed at room temperature (22–24 °C) using an Optopatch (Cairn Research Ltd, UK) amplifier. Patch pipettes were coated with surf wax (Mr Zogs SexWax, USA) to minimise the fast capacitance transient of the patch pipette. The intracellular solution of the patch pipette (2–3 MΩ) contained (in mm): Cs-glutamate, 106; CsCl, 20; MgCl_2_, 3; EGTA–CsOH, 1; Na_2_ATP, 5; Na_2_GTP, 0.3; Hepes–CsOH, 5; and sodium phosphocreatine, 10 (pH 7.3). Data acquisition was controlled by pClamp software using Digidata 1440A boards (Molecular Devices, USA). Recordings were low-pass filtered at 2.5 or 5.0 kHz (8-pole Bessel), sampled at 5 or 10 kHz and stored on computer for off-line analysis (Origin – OriginLab, USA). Membrane potentials were corrected for the residual series resistance and liquid junction potential (−11 mV).

Mechanoelectrical transducer (MET) currents were elicited by stimulating the hair bundles of OHCs using a fluid jet from a pipette (tip diameter 8–10 μm) driven by a piezoelectric disc (Johnson *et al*., 2011, 2012). The pipette tip of the fluid jet was positioned near to the bundles to elicit a maximal transducer current. Mechanical stimuli were applied as force-steps or 50-Hz sinusoids (filtered at 0.25 kHz, 8-pole Bessel) with driving voltages of ± 40 V. MET currents were recorded with a patch pipette solution containing 1 mm EGTA as the calcium buffer, which was previously assessed using perforated patch recordings (Johnson *et al*., 2008). The effect of endolymphatic Ca^2+^ concentration [reported to be between 0.02 and 0.04 mm Ca^2+^ – Bosher & Warren, 1978; Salt *et al*., 1989)] was examined by superfusing the hair bundle with 0.04 mm Ca^2+^ (buffered with 4 mm HEDTA) using a multi-barrelled pipette positioned closed to the patched cells. During the experiments in which different extracellular Ca^2+^ concentrations were used (0.04 mm or 1.3 mm), the fluid jet pipette was also filled with the same solution.

Statistical comparisons of means were made by one-way anova followed by the Tukey multiple comparison test with alpha = 0.05. Mean values are quoted ± SEM.

## Results

### Hair bundle structure – OHCs

**Fig. 2:**
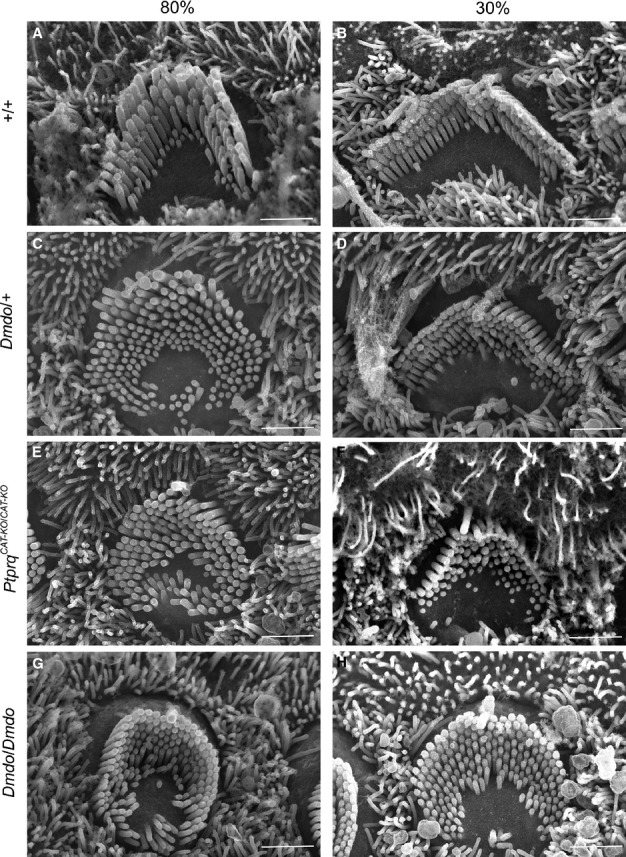
Outer hair cell stereocilia morphology at position 80% (near apex; A, C, E and G) and 30% (near base; B, D, F and H) of the length along the cochlear duct (P4) by scanning electron microscopy showed developmentally immature morphology of hair bundles in the mutants. (G and H) Diminuendo homozygotes showed the most affected hair bundles, with a rounded, almost circular shape and extra stereocilia rows. (C and D) Diminuendo heterozygotes resemble (E and F) Ptprq-CAT-KO homozygotes, being less affected but still showing differences compared to (A and B) wildtypes, such as the gently rounded hair bundle and excess microvilli. Scale bar, 1.5 μm.

**Fig. 3:**
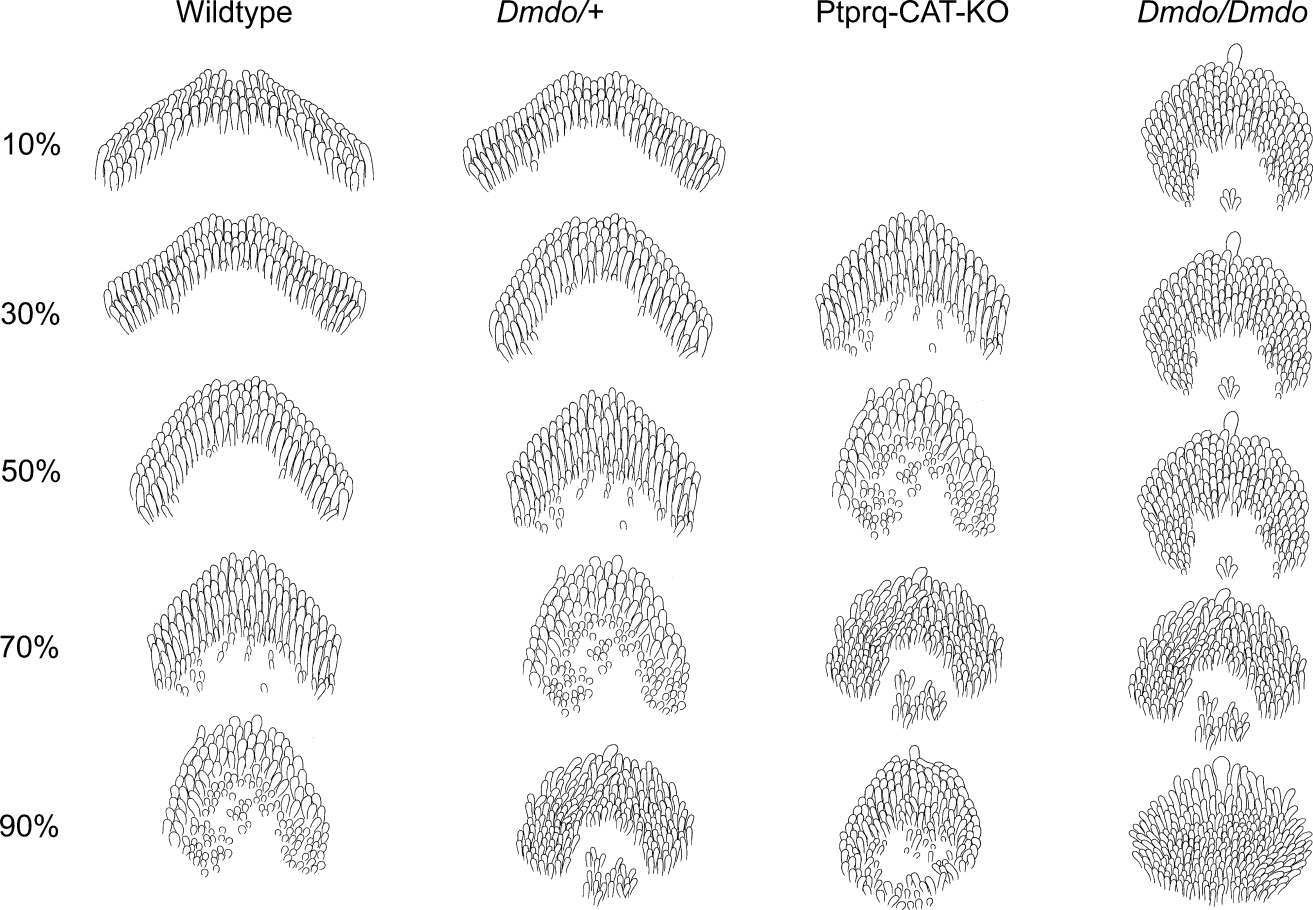
Cartoons of P4 OHCs typical of wildtype, diminuendo heterozygote, Ptprq-CAT-KO homozygote and diminuendo homozygote mice. The wildtype hair cells (left) display the typical gradient of development of outer hair cells at P4, with the most mature hair cells at the base (10%) and the most immature at the apex (90%). This gradient is still present in diminuendo heterozygote mice (centre left), but appears delayed; at each stage depicted, the hair cells resemble the next most apical stage of the wildtype. A similar difference can be observed between Ptprq-CAT-KO homozygote OHCs (centre right) and diminuendo heterozygote OHCs, with Ptprq-CAT-KO OHCs at 30% resembling diminuendo heterozygote OHCs at 50%, which resemble wildtype OHCs at 70%, for example. No Ptprq-CAT-KO OHCs were preserved for observation at 10%. Diminuendo homozygotes (right) display a much more extreme phenotype, with hair cell development appearing to stall at ∼ 50%, and hair cells more apical than that appearing very immature.

In the wildtype mice at P4, there was a gradient in maturity of OHCs from cochlear apex towards base with apical hair cell bundles showing a slightly more immature, rounded arrangement, a few short microvilli remaining within the cleft of the bundles, and narrower angles between the two arms of the V-shaped array. Basal OHCs showed a wide-angled array of stereocilia, three rows of graded height organised in straight lines with few if any excess microvilli remaining in the cleft of the V-shaped array (Figs 2A and B and 3).

All the OHCs in diminuendo homozygous mice looked immature compared with wildtype OHCs at the corresponding positions. Mutant OHCs from the base up to the 80% position showed a similar appearance, without an obvious developmental gradient (Fig. 3). In contrast, above the 80% position a developmental gradient was observed, suggesting that the development of mutant OHCs may have stalled at a set point in differentiation. The hair bundles of mutant OHCs showed a more rounded shape than those of wildtype mice and where there was evidence of a V-shaped array, the angle was narrower than that of wildtype mice. These mutant OHCs had six to eight rows of stereocilia in contrast to three rows in wildtype mice, and displayed retention of microvilli in the cleft of the bundle, with some clearly ectopic stereocilia making some bundles look more like an O-shape than a V-shape (Fig. 2H; compare to Fig. 2B).

Heterozygote (*Dmdo*/+) OHCs showed an intermediate phenotype in all notable respects. For example, they did show a developmental gradient from apex towards base, where the hair bundles had a V shape and wider angle between two arms than diminuendo homozygous mutants, but even at the base they were narrower than wildtype bundles. There were a few ectopic stereocilia at 80%, similar to diminuendo homozygous mutants, but not at 30%, where they more closely resembled wildtypes (Figs 2C and D and 3).

The Ptprq-CAT-KO homozygous OHCs showed abnormal hair bundles at P4, and we found that they appeared to share more similarities with diminuendo heterozygous OHCs than with diminuendo homozygous OHCs. First, both diminuendo heterozygote and Ptprq-CAT-KO homozygote OHCs displayed a developmental gradient from apex towards base, which seemed to be stalled in diminuendo homozygotes (Fig. 3). Second, the gross morphology of OHC bundles in diminuendo heterozygous and Ptprq-CAT-KO homozygous mice was not as rounded as diminuendo homozygous mutants (Fig. 2C–F; compare to Fig. 2G and H; see also Fig. 3). Third, there were still extra rows of stereocilia/microvilli in diminuendo heterozygotes and Ptprq-CAT-KO homozygotes, but they appeared to be fewer than in diminuendo homozygotes (Fig. 2D and F; compare to Fig. 2H). Typical OHC bundles for each genotype are summarised and drawn based on micrographs for a more direct comparison as shown in Fig. 3. The kinocilium was present in all genotypes including wildtype along the length of the cochlear duct, so was not useful as a measure of differentiation.

### Hair bundle structure – IHCs

**Fig. 4:**
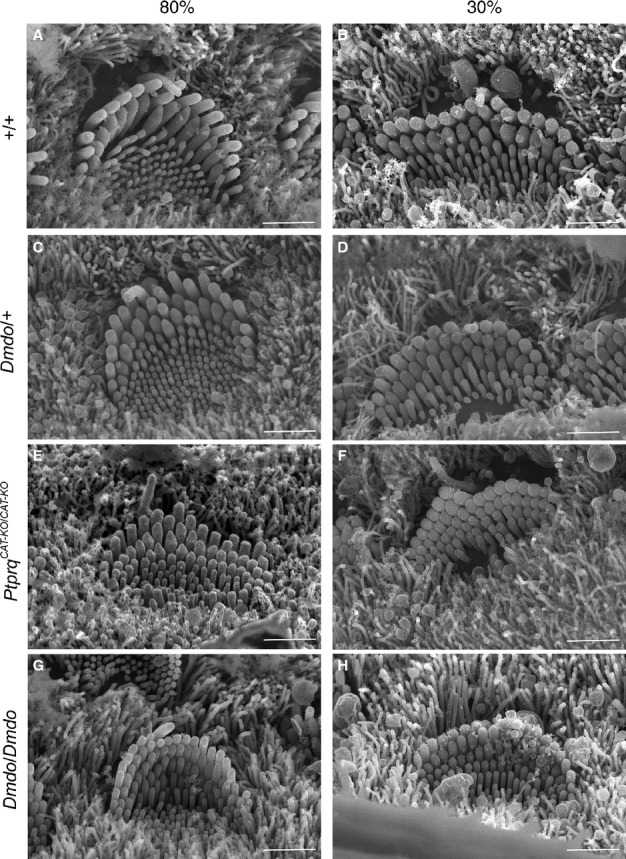
Inner hair cell stereocilia morphology at position 80% (near apex; A, C, E and G) and 30% (near base; B, D, F and H) along the length of the cochlear duct (P4) by scanning electron microscopy showed developmentally immature morphology of hair bundles in the mutants. (G and H) Diminuendo homozygote hair bundles show very little thickening of stereocilia rows. Like the outer hair cells, (C and D) diminuendo heterozygotes and (E and F) Ptprq-CAT-KO homozygotes show a less extreme phenotype but are still distinct from (A and B) the wildtype hair bundles, with an abundance of microvilli. Scale bar, 1.5 μm.

**Fig. 5:**
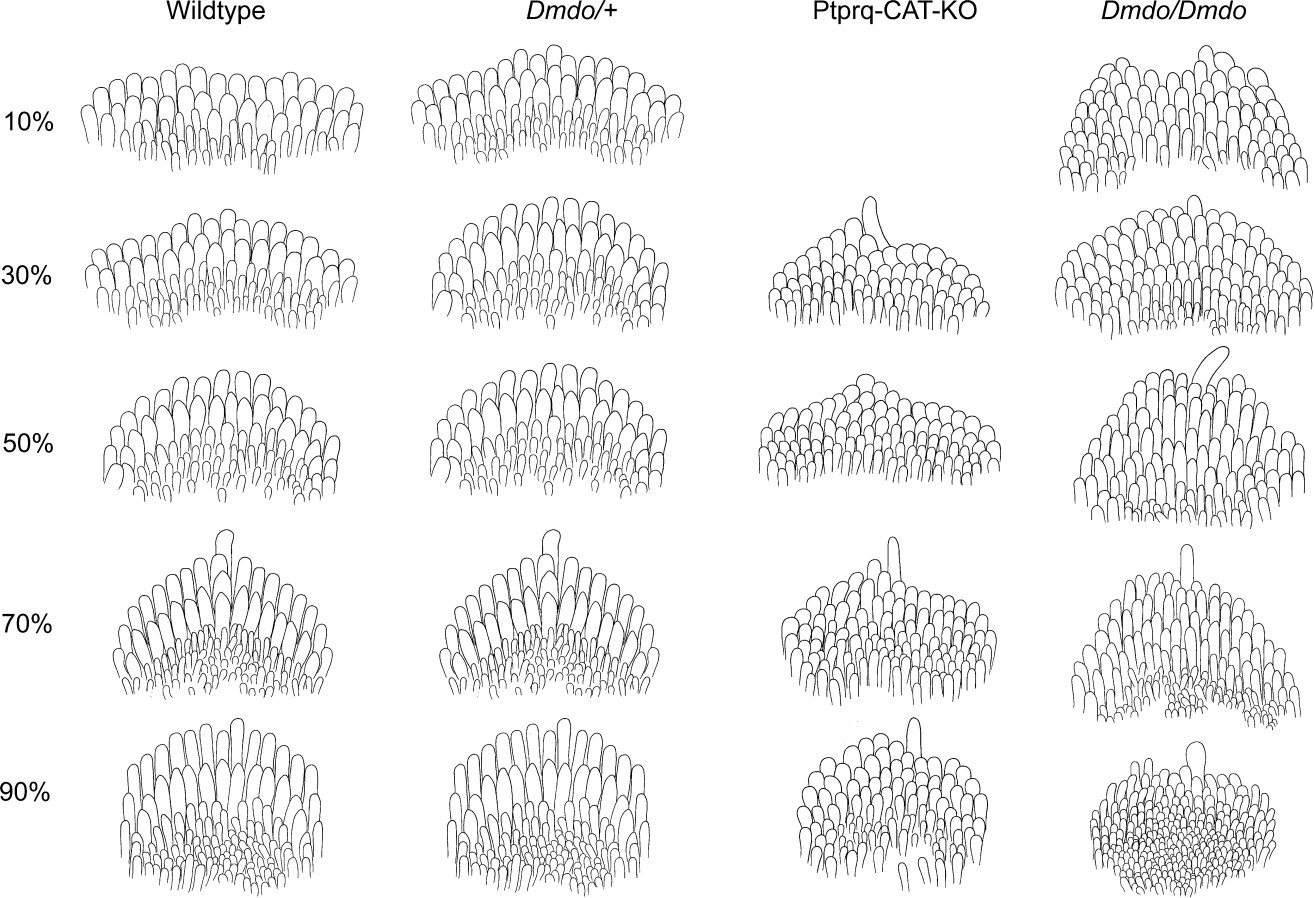
Cartoons of P4 IHCs typical of wildtype, diminuendo heterozygote, Ptprq-CAT-KO homozygote and diminuendo homozygote mice. Wildtype IHCs (left) show a gradient of maturity from the base (most mature, 10%) to the apex (least mature, 90%). Diminuendo heterozygotes (centre left) display a very similar gradient, with a slight delay in development visible from 10 to 30%. Ptprq-CAT-KO homozygote IHCs (centre right) are more affected, although stereocilia are organised into rows, and the taller stereocilia show some thickening as in wildtype and diminuendo heterozygote IHCs. No Ptprq-CAT-KO IHCs were preserved for observation at 10%. Diminuendo homozygote IHCs (right) are much more affected than the others, with extra rows of stereocilia and not much thickening of the taller stereocilia, but they still appear to have a gradient of maturity between 10 and 90%.

Most wildtype IHCs had a well-defined wide V-shape, although at the 90% position near the apex, the bundles were more rounded than further towards the base of the cochlear duct (Fig. 4A and B). IHC stereocilia had grown thicker, with the second tallest row having the thickest shafts and forming wedge-shaped (tented) apical ends, followed by the tallest row, and the innermost stereocilia were the thinnest as reported previously (Erven *et al*., 2002; Holme *et al*., 2002). Persistent microvilli were observed within the cleft of the bundle along the length of the organ of Corti. The stereocilia from the same row were almost the same height as each other, so hair bundles had a clear staircase arrangement (Figs 4A,B and 5).

**Fig. 6:**
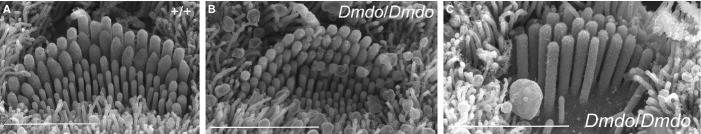
Two distinct stereocilia bundle phenotypes were observed in diminuendo homozygote inner hair cells. (A) A wildtype hair cell at 40%, showing the development of the ‘staircase’ of stereocilia heights. Microvilli are still present. (B) This is the more common diminuendo homozygote phenotype. The diminuendo homozygote hair cell resembles the wildtype but appears less mature, with still-thin stereocilia and more microvilli. (C) In contrast, a minority of homozygote inner hair cells lacked the staircase organisation and microvilli entirely. We observed no hair bundles intermediate between these two phenotypes. Scale bars, 300 μm.

IHCs of diminuendo homozygous mutants differed from those of wildtype mice mainly by not showing thickening of their stereocilia, such that there was little distinction between stereocilia rows save the height. There was also less distinction between stereocilia and microvilli than in IHCs of wildtype mice (Fig. 4G and H). A minority of homozygote inner hair cells in the basal half had hair bundles consisting of bunched stereocilia with no height gradation. No microvilli were observed in these hair cells (Fig. 6).

Diminuendo heterozygote IHCs showed an intermediate appearance but generally were much more similar to wildtype IHCs than to diminuendo homozygote IHCs (Fig. 4C and D). Heterozygote IHCs showed some thickening of stereocilia, unlike the homozygote IHCs. However, there was a slight developmental delay observed towards the base of the cochlea (Fig. 5).

The Ptprq-CAT-KO homozygous IHCs displayed a phenotype intermediate between diminuendo homozygotes and wildtypes. For example, both diminuendo heterozygote and Ptprq-CAT-KO homozygote IHCs bore extra rows of stereocilia/microvilli, but not as many as diminuendo homozygote IHCs (Fig. 4D and F; compare to Fig. 4H). Thickening of IHC stereocilia was seen in Ptprq-CAT-KO homozygous and diminuendo heterozygous mice and the arrangement of the stereocilia in the same row was neater than that of the diminuendo homozygous mice (Fig. 4C and E; compare to Fig. 4G). However, we also observed some Ptprq-CAT-KO inner hair cells resembling the diminuendo homozygote hair cells with bunched stereocilia (as shown in Fig. 6C). Typical inner hair cell bundles for each genotype are summarised and drawn based on micrographs for a more direct comparison in Fig. 5. As with OHCs, the kinocilium was observed in all genotypes and at all positions in the cochlea.

### Summary of hair cell appearance

In summary, we observed developmentally immature morphology in diminuendo heterozygous, homozygous and Ptprq-CAT-KO homozygous mice. When comparing the development of OHC hair bundles in terms of gross morphology, number of stereocilia rows and form and arrangement of stereocilia, diminuendo heterozygous and Ptprq-CAT-KO homozygous mice are more similar to each other and the morphological changes observed in these two genotypes seem not as severe as those seen in diminuendo homozygous mice. However, Ptprq-CAT-KO homozygote and diminuendo homozygote IHCs both appear markedly more affected than diminuendo heterozygote IHCs, which closely resemble wildtype IHCs (Fig. 5). The relationships can be summarised as follows:

OHCs: *Dmdo/Dmdo* < *Ptprq/Ptprq* = *Dmdo*/+ < WT
IHCs: *Dmdo/Dmdo* < *Ptprq/Ptprq* < *Dmdo*/+ < WT

These findings suggest that the downregulation of Ptprq is likely to be an important contributor to the hair cell phenotype observed in diminuendo mice, but it is not sufficient to explain all of it.

### Microarray and qRTPCR

None of the probes showed a significant difference in expression levels between wildtypes and Ptprq-CAT-KO homozygotes after significance was adjusted to allow for multiple tests. However, there were a number of interesting genes among those with an unadjusted *P* < 0.01 (Table 1). These include *Calb2*, *Hsd17b7*, *Chrna1*, *Chrna9* and *Otof*, which showed altered expression in the diminuendo microarray and further quantitative PCR, as we have previously reported (Lewis *et al*., 2009; see also Kuhn *et al*., 2011), and *Grxcr1*, which was of interest as the gene mutated in the pirouette mutant (Odeh *et al*., 2010). All six genes were downregulated in the Ptprq-CAT-KO microarray (Table 1), while in diminuendo homozygotes *Calb2*, *Chrna1*, *Chrna9* and *Hsd17b7* were downregulated (Lewis *et al*., 2009) and *Otof* was upregulated (Kuhn *et al*., 2011). These six genes were chosen for validation by quantitative RTPCR in the present study.

**Table 1:** Genes with changes in expression (unadjusted *P* < 0.01) from the microarray

Gene Name	Ensembl ID	Proportional change	*P-*value	Adjusted *P*-value
PTPRQ	ENSMUSG00000035916	−1.668142552	1.22E-05	0.258653553
RPRM	ENSMUSG00000075334	−1.42558442	0.000255653	0.999832538
ENPP2	ENSMUSG00000022425	−1.170514788	0.001266519	0.999832538
PAQR9	ENSMUSG00000064225	−1.234515233	0.001500896	0.999832538
SCARA3	ENSMUSG00000034463	−1.146440488	0.001698927	0.999832538
LRPPRC	ENSMUSG00000024120	−1.149490043	0.001915957	0.999832538
GM1027	ENSMUSG00000047502	−1.132141912	0.002083288	0.999832538
FRY	ENSMUSG00000056602	−1.316716103	0.002264682	0.999832538
TUBA1A	ENSMUSG00000072235	−1.158236745	0.002350494	0.999832538
REEP6	ENSMUSG00000035504	−1.287813751	0.002673204	0.999832538
6330409N04RIK	ENSMUSG00000021930	−1.154864691	0.00278719	0.999832538
GRXCR1	ENSMUSG00000068082	−1.374271986	0.003023447	0.999832538
HAUS1	ENSMUSG00000041840	−1.460383181	0.00302821	0.999832538
CXADR	ENSMUSG00000022865	−1.177070915	0.003301987	0.999832538
CALB2	ENSMUSG00000003657	−1.872545244	0.003340481	0.999832538
HS6ST2	ENSMUSG00000062184	−1.197160378	0.003409697	0.999832538
HSD17B7	ENSMUSG00000026675	−1.508593767	0.003609971	0.999832538
TCEA3	ENSMUSG00000001604	1.145237128	0.003669075	0.999832538
CYLD	ENSMUSG00000036712	−1.221211996	0.003802919	0.999832538
FAM177a	ENSMUSG00000095595	−1.298189092	0.003856988	0.999832538
GHSR	ENSMUSG00000051136	−1.133219164	0.003930565	0.999832538
MYCL1	ENSMUSG00000028654	−1.455969357	0.005057643	0.999832538
CXADR	ENSMUSG00000022865	−1.194386586	0.005408051	0.999832538
HSPA2	ENSMUSG00000059970	−1.32492769	0.005413211	0.999832538
ACCN2	ENSMUSG00000023017	−1.223432756	0.005542887	0.999832538
CYLD	ENSMUSG00000036712	−1.163235475	0.005846245	0.999832538
ASAP2	ENSMUSG00000052632	1.119392959	0.006286889	0.999832538
ANO1	ENSMUSG00000031075	−1.408344537	0.006989795	0.999832538
SLC25A5	ENSMUSG00000016319	−1.108047476	0.007157786	0.999832538
MARVELD3	ENSMUSG00000001672	−1.243287418	0.007360472	0.999832538
6030405A18Rik	ENSMUSG00000056306	−1.366002261	0.007580547	0.999832538
TMEM25	ENSMUSG00000002032	−2.539909188	0.007733011	0.999832538
C030014I23RIK	ENSMUSG00000060380	−1.291455753	0.0077525	0.999832538
NFE2L3	ENSMUSG00000029832	−1.251621116	0.007866223	0.999832538
KCTD3	ENSMUSG00000026608	−1.101026278	0.008013213	0.999832538
AFTPH	ENSMUSG00000049659	−1.153614005	0.008310229	0.999832538
KIF2A	ENSMUSG00000021693	−1.131059659	0.008322974	0.999832538
PRKCDBP	ENSMUSG00000037060	1.181724169	0.008391717	0.999832538
2810011L19RIK	ENSMUSG00000097929	−1.639169358	0.008672408	0.999832538
ANGPTL2	ENSMUSG00000004105	1.173678608	0.008928208	0.999832538
C030014I23RIK	ENSMUSG00000060380	−1.296314718	0.008978949	0.999832538
CHRNA1	ENSMUSG00000027107	−1.689431657	0.009032761	0.999832538
SEMA3E	ENSMUSG00000063531	−1.288802355	0.009075836	0.999832538
ERLIN2	ENSMUSG00000031483	−1.137591058	0.009078345	0.999832538
OTOF	ENSMUSG00000062372	−1.822630656	0.009080105	0.999832538
HDAC2	ENSMUSG00000019777	−1.155381566	0.009307828	0.999832538
KCTD14	ENSMUSG00000051727	1.133454846	0.00948616	0.999832538
LRRC24	ENSMUSG00000033707	−1.173728732	0.009519745	0.999832538
TLN2	ENSMUSG00000052698	1.151209002	0.00963685	0.999832538
CHRNA9	ENSMUSG00000029205	−1.462315299	0.009711404	0.999832538

List of genes from the microarray with differences in expression between Pprq-CAT-KO homozygotes and wildtype littermates (unadjusted *P* < 0.01). Four genes are known to be involved in deafness (*PTPRQ, OTOF, GRXCR1* and *CHRNA9*).

**Fig. 7:**
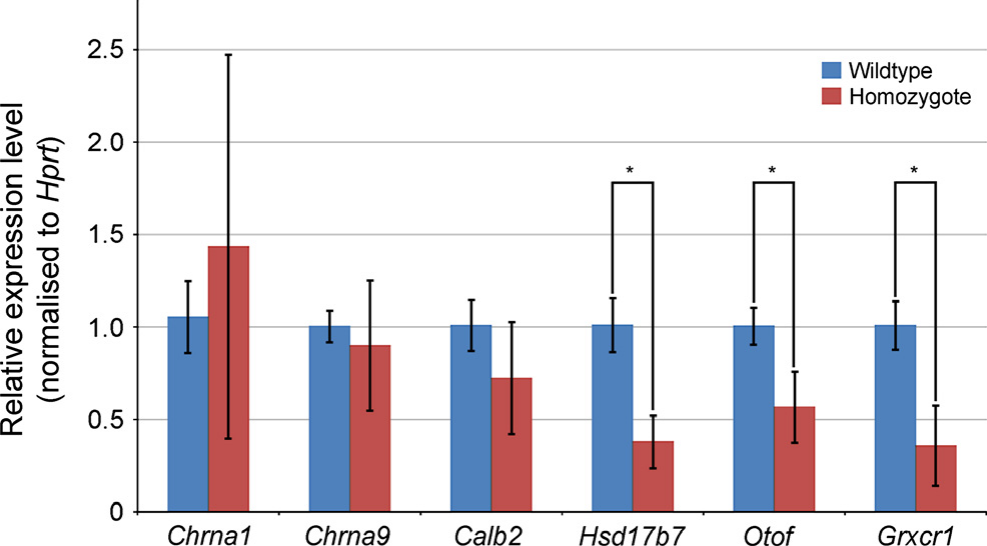
Quantitative real time PCR on cDNA from organ of Corti RNA from Ptprq-CAT-KO homozygote and wildtype littermates at P4. Three pairs were used for each comparison. Quantities have been normalised to *Hprt* levels, and *Jag1* was used to control for sensory tissue; all pairs used showed no significant difference in expression of *Jag1*. *Hsd17b7*, *Otof* and *Grxcr1* are all significantly downregulated in Ptprq-CAT-KO homozygotes. *P < 0.01. Error bars represent SD.

qRTPCR of these six genes showed that *Grxcr1*, *Otof* and *Hsd17b7* were all significantly downregulated in Ptprq-CAT-KO homozygotes compared to wildtype littermates [littermates *Grxcr1*, *P* = 1.72 × 10^−7^(Student's *t*-test); *Otof*, *P* = 4.52 × 10^−5^(Welch's *t*-test); *Hsd17b7*, *P* = 7.52 × 10^−9^(Student's *t*-test)] ((Fig. 7), confirming the microarray results. The expression levels of *Chrna1* in Ptprq-CAT-KO homozygotes relative to wildtype littermates varied widely between the three pairs used, and *Chrna9* and *Calb2* were not significantly affected [*Chrna9*, *P* = 1 (Wilcoxon *t*-test); *Calb2*, *P* = 0.15 (Welch's *t*-test)]. Expression of *Grxcr1* was not significantly altered in the diminuendo microarray and *Otof* is upregulated in diminuendo homozygotes (Lewis *et al*., 2009; Kuhn *et al*., 2011), so in this case only the *Hsd17b7* downregulation resembles the diminuendo homozygote phenotype.

**Fig. 8:**
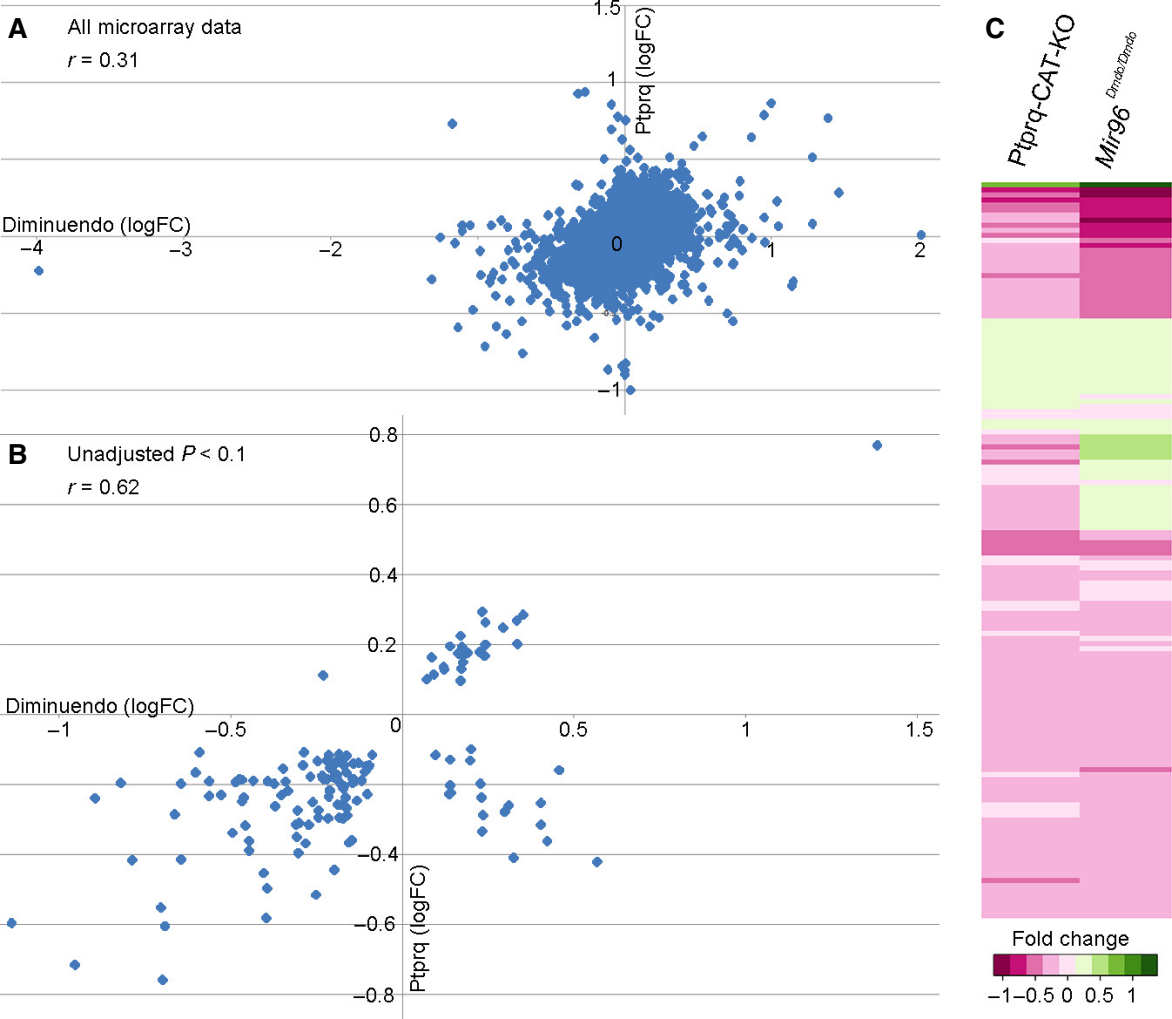
(A and B) Scattergraphs showing correlation of data from the two microarrays; A shows the correlation of all genes present in both microarrays regardless of significance while B shows the correlation of those genes with an unadjusted *P* < 0.1 in both microarrays. (C) Heatmap created using the R statistical software package, showing genes with an unadjusted *P* < 0.1 from the Ptprq-CAT-KO microarray (left) compared with the same genes (also with unadjusted *P* < 0.1) from the diminuendo microarray (right). Genes are compared and coloured according to their proportional change, on a scale from dark pink (most downregulated) to dark green (most upregulated).

We also compared the microarray data on a larger scale, using first a Pearson correlation comparing the probe log proportional changes across both microarrays (13 542 of the probes were present in both microarrays, out of 21 229 in the Ptprq microarray and 25 044 in the diminuendo array). This resulted in a Pearson coefficient of 0.31, which is a comparatively low correlation (Fig. 8A). When considering only probes with an unadjusted *P*-value < 0.1 in both arrays (146 probes, of 1012 probes with *P* < 0.1 in the Ptprq microarray and 2506 probes with *P* < 0.1 in the diminuendo microarray), however, the coefficient was 0.62 (Fig. 8B). The same data (probes with unadjusted *P* < 0.1 from both microarrays) were used to create a heatmap visualisation of the probe proportional changes from both arrays (Fig. 8C). While the magnitude of regulation differed between diminuendo homozygotes and Ptprq-CAT-KO homozygotes, the scattergraph and heatmap show that most of the genes were regulated similarly; those upregulated in diminuendo homozygotes were upregulated in Ptprq-CAT-KO homozygotes (green in the heatmap) and those downregulated in diminuendo homozygotes were downregulated in Ptprq-CAT-KO homozygotes (pink in the heatmap). The magnitude of up- or downregulation compared to wildtype littermates was noticeably greater in the diminuendo array than in the Ptprq-CAT-KO array (indicated by the darker colours).

**Fig. 9:**
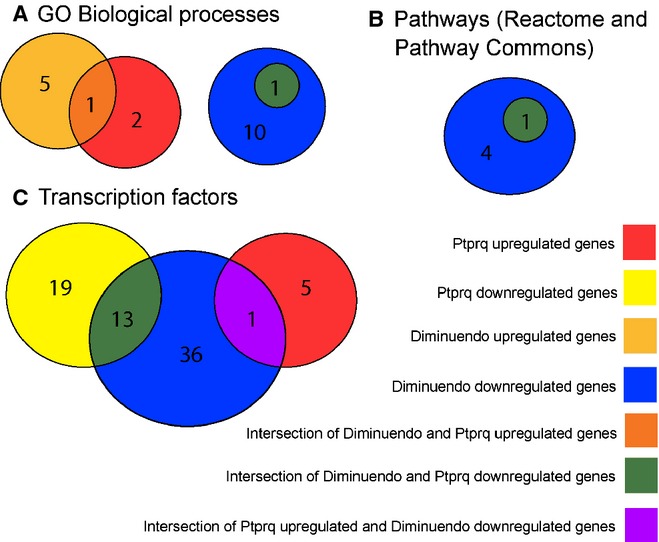
Venn diagrams showing the numbers of gene sets significantly enriched in the diminuendo and Ptprq microarrays, and how many are common to both. (A) Gene sets annotated with GO biological processes (from the mSigDB dataset); (B) gene sets defined by Pathway Commons and Reactome corresponding to known biological pathways; (C) gene sets regulated by transcription factors (from the mSigDB dataset).

Pathway analysis was carried out using GSEA with ranked lists of genes from both arrays; again, an unadjusted *P* < 0.1 was used. GSEA takes a list of genes with a ranking (in this case, the log of the proportional change) and a set of known pathways and tests to see whether any of the pathways are over-represented by the genes at the top or the bottom of the ranked list. Overall, we found more pathways significantly over-represented in diminuendo dysregulated genes (*P* < 0.05) but, after removing uninformative pathways (such as ‘system development’ and ‘system process’), several pathways were significantly enriched in both Ptprq and diminuendo dysregulated genes (Fig. 9A and B, Table 2). The shared pathways related to the neurological system and to cell proliferation. No canonical pathways (as defined by mSigDB) were shared, but there were multiple transcription factors implicated in the transcriptional phenotype of both mutants (Fig. 9C).

**Table 2:** Details of pathway enrichment in the diminuendo and Ptprq microarrays

	Diminuendo genes		Ptprq genes	
		
	Upregulated	Downregulated	Upregulated	Downregulated
GO Biological Processes	6 (76)	17 (103)	3 (30)	2 (64)
Reactome	9 (55)	4 (73)	1 (9)	1 (19)
Pathway Commons	0 (22)	2 (124)	1 (11)	1 (68)
Transcription factors	3 (114)	55 (430)	6 (85)	38 (336)

Genes with unadjusted *P* < 0.1 were used, compared to four different gene sets – genes annotated with GO Biological Processes, genes playing a role in Reactome pathways, genes associated with Pathway Commons pathways and genes regulated by transcription factor-associated regulatory sites. The number of significantly enriched pathways (*P* < 0.05) is followed by the total number of gene sets considered in brackets. In order for a gene set to be considered, at least ten of its genes had to be present in the list compiled from the microarray.

The broad pattern of the transcriptome of Ptprq-CAT-KO homozygotes resembled that of diminuendo homozygotes but was not as severe. In addition, not all the genes affected were regulated in the same direction. The downregulation of Ptprq may contribute to but cannot explain all the transcriptome effects seen in diminuendo mice.

### Hair cell transducer current is impaired in diminuendo mutant mice

**Fig. 10:**
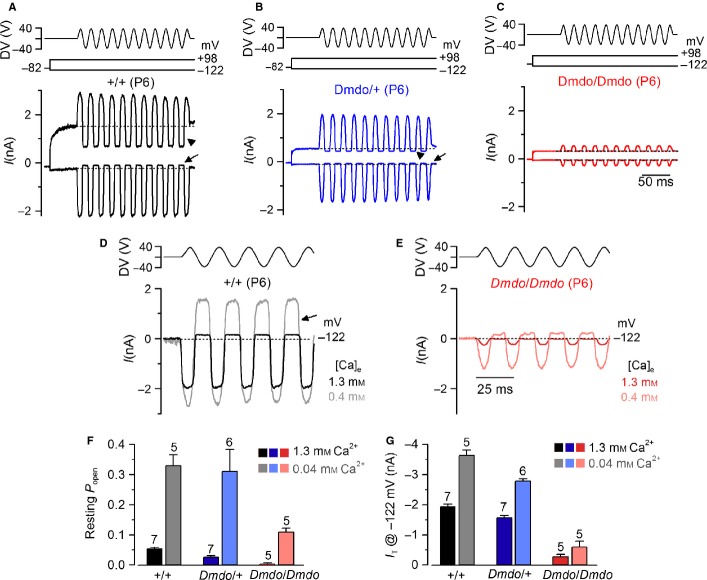
Mechanotransducer currents in diminuendo cochlear outer hair cells. (A–C) Saturating transducer currents recorded from (A) a control (B), a heterozygous and (C) a homozygous mutant P6 apical-coil diminuendo OHC by applying sinusoidal force stimuli of 50 Hz to the hair bundles at −122 mV and + 98 mV. The driver voltage (DV) signal of ± 40 V to the fluid jet is shown above the traces (negative deflections of the DV are inhibitory). The holding potential was −82 mV. Extracellular Ca^2+^ concentration was 1.3 mm. The arrows and arrowheads indicate the closure of the transducer currents (i.e. resting current) elicited during inhibitory bundle displacements at hyperpolarized and depolarized membrane potentials, respectively. Note that the resting current increases with membrane depolarization. Dashed lines indicate the holding current, which is the current at the holding membrane potential. (D and E) Comparison of transducer currents recorded from (D) a control and (E) a homozygous mutant P6 diminuendo OHC in the presence of 1.3 mm Ca^2+^ (black/red) and endolymphatic-like Ca^2+^ concentration (0.04 mm; grey/pink lines) at −122 mV. (F and G) Resting current (F) and (G) peak transducer current at a membrane potential of −122 mV recorded in OHCs from the three genotypes in the presence of 1.3 mm (black/blue/red) and 0.04 mm (grey/pale blue/pink) extracellular Ca^2+^.

We measured MET currents from OHCs of diminuendo mice in order to compare them with those previously recorded from cells of Ptprq-mutant mice, and they showed a reduced amplitude (Goodyear *et al*., 2003). Transducer currents from P6 apical-coil OHCs in diminuendo control (+/+), heterozygous (*Dmdo*/+) and homozygous mutant (*Dmdo/Dmdo*) mice were elicited by alternating inhibitory and excitatory bundle displacements using 50-Hz sinusoidal force stimuli (Johnson *et al*., 2011, 2012). Upon moving the bundles in the excitatory direction (i.e. towards the taller stereocilia) and at negative membrane potentials, an inward MET current could be elicited in OHCs from all genotypes (Fig. 10A–C), suggesting that the tip links were present and functional. The maximal MET current in control OHCs (−1941 ± 79 pA at −122 mV, *n* = 7) was significantly larger than that in heterozygous (−1581 ± 68 pA, *n* = 6, one-way anova, Tukey post-test – *P* < 0.01) and homozygous (−273 ± 78 pA *n* = 5, one-way anova, Tukey post-test – *P* < 0.0001) mutant cells (overall significance – *F*_2,15_ = 123.1, *P* < 0.0001). Any resting current flowing through open MET channels in the absence of mechanical stimulation was reduced when bundles were moved in the inhibitory direction (i.e. away from the taller stereocilia) in all control and mutant OHCs (Fig. 10A–C, arrows). However, the open probability of MET channels at rest in 1.3-mm Ca^2+^ was significantly larger in control (0.055 ± 0.004 at −122 mV, *n* = 7) than in heterozygous (0.026 ± 0.005 pA, *n* = 6, one-way anova, Tukey post-test – *P* < 0.001) and homozygous (0.004 ± 0.003 pA, *n* = 5, one-way anova, Tukey post-test – *P* < 0.0001) mutant cells (overall significance – *F*_2,15_ = 33.5, *P* < 0.0001). Because the MET current reverses near 0 mV (Zampini *et al*., 2011), it became outward when excitatory bundle stimulation was applied during voltage steps positive to its reversal potential (Fig. 10A–C). At positive potentials (+ 98 mV), the larger resting transducer current (Fig. 10A–C, arrowheads) is likely to be due to an increased open probability of the transducer channel resulting from a reduced driving force for Ca^2+^ influx (Crawford *et al*., 1989; Johnson *et al*., 2011). Note that the residual outward K^+^ current observed at + 98 mV, just before the beginning of the MET current, was largely reduced in heterozygous and homozygous mutant OHCs, which is consistent with a failure in the normal development of the basolateral membrane currents in these diminuendo mutant cells (Kuhn *et al*., 2011). We asked whether the Ca^2+^ sensitivity of the MET current was affected in diminuendo mutant OHCs by locally superfusing their hair bundle with a solution containing an endolymph-like concentration of Ca^2+^ (0.04 mm; Bosher & Warren, 1978). Lowering the extracellular Ca^2+^ concentration is known to increase both the maximum MET current and its fraction activated at rest. Calcium is a permeant blocker of the transducer channel (Ricci & Fettiplace, 1998; Marcotti *et al*., 2005), so the increased current amplitude in low Ca^2+^ is caused by the partial relief of this block. Moreover, extracellular Ca^2+^ causes adaptation and as such closes some transducer channels. Therefore, reducing Ca^2+^ influx into the transducer channel, by either depolarizing hair cells to near the Ca^2+^ equilibrium potential (as shown in Fig. 10A–C) or lowering the extracellular concentration, causes an increased open probability of the channel (Crawford *et al*., 1991). In diminuendo mice, both phenomena were observed because decreasing the Ca^2+^ concentration from 1.3 to 0.04 mm increased the resting (Fig. 10D–F) and maximal (Fig. 10D, E and G) MET current in control and homozygous OHCs. The above results indicate that the size of the MET current, but not its Ca^2+^ sensitivity, is affected by mutation in miR-96.

**Fig. 11:**
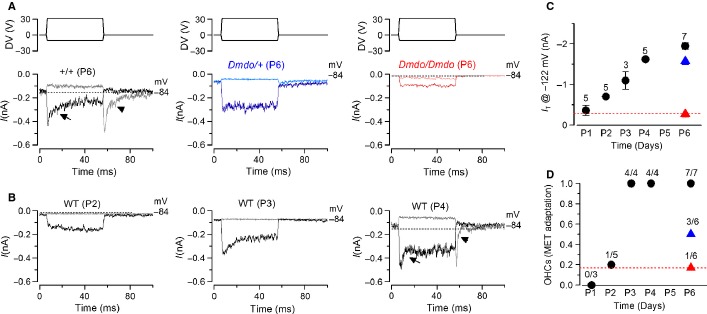
The development of transducer currents in diminuendo mutant OHCs is prematurely arrested. (A) Driver voltages to the fluid jet (top panels) and transducer currents recorded at −84 mV from a control, a heterozygous and a homozygous diminuendo mutant P6 OHC. Positive driver voltages (excitatory direction) elicited inward (negative) transducer currents that declined or adapted over time only in control OHCs (arrow). A small transducer current was present at rest (before *t* = 0) and inhibitory bundle displacement turned this off. Upon termination of the inhibitory stimulus, the transducer current in control OHCs showed evidence of rebound adaptation (arrowhead). (B) Example of transducer currents in developing control OHCs (P2, P3 and P4). Note that both signs of current adaptation (arrow and arrowhead) are first detected at P4. (C) Maximal transducer current recorded at different postnatal ages in control OHCs (black) and that of the heterozygous (blue) and homozygous (red) diminuendo mutant cells at P6. The number of OHCs tested is shown above each data point. (D) Extent of adaptation for small bundle deflection towards the excitatory directions (see arrows in panel A, left panel) of the transducer current at different postnatal ages in control OHCs (black) and that of the heterozygous (blue) and homozygous (red) diminuendo mutant cells at P6. The number of OHCs showing MET current adaptation over the total number of cells tested is shown above each data point. Note that (C) the size and (D) the extent of adaptation recorded in P6 homozygous diminuendo OHCs is similar to that measured in P1–2 control cells.

miR-96 has recently been shown to regulate the progression of the physiological differentiation of cochlear hair cells as in its absence the development of the basolateral biophysical properties is arrested just after the day of birth (Kuhn *et al*., 2011). We tested whether miR-96 was also able to control the normal developmental maturation of the mechanoelectrical transduction apparatus in hair cells by comparing the properties of the MET current in heterozygous and homozygous diminuendo OHCs to that of control cells. Hair bundles from OHCs were stimulated using alternating inhibitory and excitatory step mechanical stimuli instead of sinusoids, which allows the measurement of MET channel adaptation in addition to current size. In control P6 OHCs, excitatory bundle movements with non-saturating stimuli elicited rapid inward currents at a holding potential of −84 mV (Fig. 11A, arrow in left panel). Inhibitory hair bundle stimulation shuts off the small fraction of current flowing at rest (see also Fig. 10A) and, at the offset of large inhibitory steps, a transient rebound (downward dip; Fig. 11A, arrowhead in left panel) was observed. All these manifestations of MET current adaptation are similar to those described in control auditory hair cells from lower vertebrates and mice (Crawford *et al*., 1989; Kennedy *et al*., 2005). Slipping and rebound adaptation were largely reduced or absent in heterozygous and homozygous diminuendo P6 OHCs (Fig. 11A, middle and right panels). We then compared the MET current responses in diminuendo mutant OHCs (Fig. 11A) with those recorded in control cells during early postnatal development (P1–6; Fig. 11B shows examples at P2, P3 and P4). We found that both the amplitude (Fig. 11C) and the extent (Fig. 11D) of adaptation during excitatory stimulation (Fig. 11A, arrow in left panel) of the MET current in P6 OHCs from diminuendo homozygous mutant were similar to those recorded in P1-P2 control cells (Fig. 11C and D, respectively; red). An intermediate phenotype was seen for heterozygous diminuendo OHCs (Fig. 11C and D; blue).

In summary, we have shown that the biophysical properties of the MET currents in diminuendo homozygous OHCs are arrested at a very early stage of postnatal development. We also see a strong similarity between the reduced amplitude of the MET current in diminuendo heterozygotes with that previously reported in OHCs from Ptprq-null mutant mice (Goodyear *et al*., 2003). However, both slipping and rebound adaptation are absent or largely reduced in diminuendo heterozygotes while they were still present in *Ptprq*-null mutants (Goodyear *et al*., 2003).

## Discussion

Homozygous diminuendo mice at P4 lack the normal gradient of hair bundle development of OHCs between 0 and 80% of the cochlear length while above that in the apex the hair cells are still less mature than those at the equivalent position in wildtype littermates. This suggests developmental retardation followed by stalling at a certain point in differentiation. The hair cells appear unable to differentiate any further. Heterozygous diminuendo mice show a delay in differentiation of OHCs at P4 but there is no stalling of development at this stage; the gradient of maturity is present throughout the organ of Corti. We have previously shown that, at 4 weeks old, diminuendo heterozygote OHCs still display significantly narrower bundle span than wildtypes while supporting cells have wider apices, and hair cell density remains the same (see Supporting Information Fig. s1p and q in Lewis *et al*., 2009). The OHCs of P4 Ptprq-CAT-KO homozygous mice also appear to exhibit delayed differentiation, similar to diminuendo heterozygotes but with a greater delay (Fig. 3). The IHCs of diminuendo heterozygotes are broadly similar to wildtypes, but both diminuendo homozygote and Ptprq-CAT-KO homozygote IHCs have disorganised stereocilia bundles. However, Ptprq-CAT-KO IHCs display more hallmarks of maturity than diminuendo homozygote IHCs, such as well-organised stereocilia rows and thickening of stereocilia tips. The overall phenotype of Ptprq-CAT-KO hair cells bears a striking resemblance to that of diminuendo heterozygous mice and is not as severe as that of diminuendo homozygous mice, suggesting that the reduction in Ptprq activity is a major contributor to the diminuendo stereocilia morphological phenotype. This observation also fits with part of the electrophysiological phenotype, where heterozygous diminuendo mice resemble homozygous *Ptprq*-null mice (Goodyear *et al*., 2003), with a reduced MET current amplitude compared to controls. In diminuendo homozygotes the phenotype is more severe, as normal development of the MET current amplitude appears to be arrested just after birth (P1–2), consistent with the proposed role of miR-96 in regulating the progression of the physiological and morphological differentiation of mammalian cochlear hair cells (Kuhn *et al*., 2011). Furthermore, the downregulation of *Ptprq* expression in the diminuendo mutants is not likely to be responsible for the adaptation defects seen in these mice, because adaptation in *Ptprq* mutants is near to normal (Goodyear *et al*., 2003).

The transcriptome analyses, on the other hand, show less of a similarity between Ptprq-CAT-KO and diminuendo. This is not unexpected – Ptprq is not a regulatory factor but performs a specific function in the hair cell, while miR-96, which is a regulator, would be expected to affect the expression of many genes. However, when just the genes with a *P*-value < 0.1 are considered, there are similarities between Ptprq-CAT-KO and diminuendo, with many of the genes affected in Ptprq-CAT-KO homozygotes being similarly regulated in diminuendo homozygotes, as seen in the heatmap (Fig. 8C) and reflected in the correlation coefficient, *r* = 0.62 (Fig. 8B). An initial exploration of pathways affected shows that some of the pathways and transcription factors implicated by the transcriptional data are common to both mutants. Of the three genes whose expression changes were confirmed by qRTPCR, however, only downregulation of *Hsd17b7* is seen in both Ptprq-CAT-KO and diminuendo homozygotes. *Grxcr1* is not significantly affected in diminuendo homozygotes, and *Otof* is upregulated in diminuendo homozygotes and downregulated in Ptprq-CAT-KO homozygotes (Lewis *et al*., 2009; Kuhn *et al*., 2011). In addition, many of the other major molecular changes observed in diminuendo homozygotes are not reproduced in Ptprq-CAT-KO homozygotes, for example the downregulation of *Ocm*. It is possible that *Hsd17b7* is downregulated in both mutants because of a specific effect for which lack of Ptprq is responsible. *Hsd17b7* (hydroxysteroid (17-beta) dehydrogenase 7) is involved in cholesterol biosynthesis and is required for neuronal survival early in development; mice homozygous for a disrupted allele of *Hsd17b7* die at embryonic day 10.5, with increased apoptosis observed in the neural tube, dorsal root ganglia and trigeminal nerve. They also display cardiovascular defects (Shehu *et al*., 2008; Jokela *et al*., 2010). Mice with an ENU-induced mutation in *Hsd17b7* display disrupted Hedgehog signalling. The mutation is hypomorphic; it leads to most of the *Hsd17b7* transcripts being truncated due to a mis-splice, but low levels of wildtype transcript are still present (Stottmann *et al*., 2011). Hedgehog signalling is required for inner ear development (Liu *et al*., 2002; Riccomagno *et al*., 2002), but any more direct link between Ptprq and Hsd17b7 remains unclear. The changes in expression of *Grxcr1* in Ptprq-CAT-KO homozygotes, and of *Otof* in both diminuendo and Ptprq-CAT-KO homozygotes, may be only indicative of a dysfunctional hair cell.

In conclusion, many of the morphological and electrophysiological features of diminuendo heterozygotes are reproduced in Ptprq-CAT-KO homozygotes, and in some features the transcriptome of Ptprq-CAT-KO homozygotes resembles that of diminuendo homozygotes. It is particularly interesting that in the P4 diminuendo cochlea there is very little difference in expression of *Ptprq* between heterozygotes and homozygotes; *Ptprq* is downregulated to ∼ 62% of the wildtype level in heterozygotes and ∼56% of the wildtype level in homozygotes (Lewis *et al*., 2009), which would suggest that diminuendo heterozygotes and homozygotes should both resemble Ptprq heterozygotes, which is not the case; Ptprq-CAT-KO heterozygotes have no hair cell defects (Goodyear *et al*., 2003). The reduction in *Ptprq* seen in diminuendo mice appears to be a major contributor to the phenotype, particularly the apparent delay and eventual stalling of hair cell development observed by scanning electron microscopy and the delayed development of the MET current, but it does not account for all of the observations. The more severe phenotypes in the diminuendo homozygous mice indicate that other genes besides Ptprq are contributing to the diminuendo homozygous phenotype, and these genes may not be affected in diminuendo heterozygous or Ptprq-null mice.

## Abbreviations

GSEA: Gene Set Enrichment Analysis (software)
IHC: inner hair cell
MET: mechanoelectrical transducer (current)
OHC: outer hair cell
P: postnatal day
Ptprq-CAT-KO: a catalytically inactive form of Ptprq

## References

[b100] Benjamini Y, Hochberg Y (1995). Controlling the false discovery rate: a practical and powerful approach to multiple testing. J. R. Stat. Soc. Ser. B.

[b1] Bolstad B (2001). http://bmbolstad.com/stuff/qnorm.pdf.

[b2] Bolstad BM, Irizarry RA, Astrand M, Speed TP (2003). A comparison of normalization methods for high density oligonucleotide array data based on variance and bias. Bioinformatics.

[b3] Bosher SK, Warren RL (1978). Very low calcium content of cochlear endolymph, an extracellular fluid. Nature.

[b4] Chen J, Ingham N, Clare S, Raisen C, Vancollie VE, Ismail O, McIntyre RE, Tsang SH, Mahajan VB, Dougan G, Adams DJ, White JK, Steel KP (2013). Mcph1-deficient mice reveal a role for MCPH1 in otitis media. PLoS ONE.

[b5] Crawford AC, Evans MG, Fettiplace R (1989). Activation and adaptation of transducer currents in turtle hair cells. J. Physiol.

[b6] Crawford AC, Evans MG, Fettiplace R (1991). The actions of calcium on the mechano-electrical transducer current of turtle hair cells. J. Physiol.

[b7] Erven A, Skynner MJ, Okumura K, Takebayashi S, Brown SD, Steel KP, Allen ND (2002). A novel stereocilia defect in sensory hair cells of the deaf mouse mutant Tasmanian devil. Eur. J. Neurosci.

[b8] Gentleman RC, Carey VJ, Bates DM, Bolstad B, Dettling M, Dudoit S, Ellis B, Gautier L, Ge Y, Gentry J, Hornik K, Hothorn T, Huber W, Iacus S, Irizarry R, Leisch F, Li C, Maechler M, Rossini AJ, Sawitzki G, Smith C, Smyth G, Tierney L, Yang JY, Zhang J (2004). Bioconductor: open software development for computational biology and bioinformatics. Genome Biol.

[b9] Goodyear RJ, Legan PK, Wright MB, Marcotti W, Oganesian A, Coats SA, Booth CJ, Kros CJ, Seifert RA, Bowen-Pope DF, Richardson GP (2003). A receptor-like inositol lipid phosphatase is required for the maturation of developing cochlear hair bundles. J. Neurosci.

[b10] Goodyear RJ, Jones SM, Sharifi L, Forge A, Richardson GP (2012). Hair bundle defects and loss of function in the vestibular end organs of mice lacking the receptor-like inositol lipid phosphatase PTPRQ. J. Neurosci.

[b11] Holme RH, Kiernan BW, Brown SD, Steel KP (2002). Elongation of hair cell stereocilia is defective in the mouse mutant whirler. J. Comp. Neurol.

[b12] Hunter-Duvar IM (1978). A technique for preparation of cochlear specimens for assessment with the scanning electron microscope. Acta Otolaryngol. Suppl.

[b13] Johnson SL, Forge A, Knipper M, Munkner S, Marcotti W (2008). Tonotopic variation in the calcium dependence of neurotransmitter release and vesicle pool replenishment at mammalian auditory ribbon synapses. J. Neurosci.

[b14] Johnson SL, Beurg M, Marcotti W, Fettiplace R (2011). Prestin-driven cochlear amplification is not limited by the outer hair cell membrane time constant. Neuron.

[b15] Johnson SL, Kennedy HJ, Holley MC, Fettiplace R, Marcotti W (2012). The resting transducer current drives spontaneous activity in prehearing mammalian cochlear inner hair cells. J. Neurosci.

[b16] Jokela H, Rantakari P, Lamminen T, Strauss L, Ola R, Mutka AL, Gylling H, Miettinen T, Pakarinen P, Sainio K, Poutanen M (2010). Hydroxysteroid (17beta) dehydrogenase 7 activity is essential for fetal de novo cholesterol synthesis and for neuroectodermal survival and cardiovascular differentiation in early mouse embryos. Endocrinology.

[b17] Kennedy HJ, Crawford AC, Fettiplace R (2005). Force generation by mammalian hair bundles supports a role in cochlear amplification. Nature.

[b18] Kuhn S, Johnson SL, Furness DN, Chen J, Ingham N, Hilton JM, Steffes G, Lewis MA, Zampini V, Hackney CM, Masetto S, Holley MC, Steel KP, Marcotti W (2011). miR-96 regulates the progression of differentiation in mammalian cochlear inner and outer hair cells. Proc. Natl. Acad. Sci. USA.

[b19] Lewis MA, Quint E, Glazier AM, Fuchs H, De Angelis MH, Langford C, van Dongen S, Abreu-Goodger C, Piipari M, Redshaw N, Dalmay T, Moreno-Pelayo MA, Enright AJ, Steel KP (2009). An ENU-induced mutation of miR-96 associated with progressive hearing loss in mice. Nat. Genet.

[b20] Liu W, Li G, Chien JS, Raft S, Zhang H, Chiang C, Frenz DA (2002). Sonic hedgehog regulates otic capsule chondrogenesis and inner ear development in the mouse embryo. Dev. Biol.

[b21] Livak KJ, Schmittgen TD (2001). Analysis of relative gene expression data using real-time quantitative PCR and the 2(-Delta Delta C(T)) Method. Methods.

[b22] Marcotti W, van Netten SM, Kros CJ (2005). The aminoglycoside antibiotic dihydrostreptomycin rapidly enters mouse outer hair cells through the mechano-electrical transducer channels. J. Physiol.

[b23] Mencia A, Modamio-Hoybjor S, Redshaw N, Morin M, Mayo-Merino F, Olavarrieta L, Aguirre LA, del Castillo I, Steel KP, Dalmay T, Moreno F, Moreno-Pelayo MA (2009). Mutations in the seed region of human miR-96 are responsible for nonsyndromic progressive hearing loss. Nat. Genet.

[b24] Mootha VK, Lindgren CM, Eriksson KF, Subramanian A, Sihag S, Lehar J, Puigserver P, Carlsson E, Ridderstrale M, Laurila E, Houstis N, Daly MJ, Patterson N, Mesirov JP, Golub TR, Tamayo P, Spiegelman B, Lander ES, Hirschhorn JN, Altshuler D, Groop LC (2003). PGC-1alpha-responsive genes involved in oxidative phosphorylation are coordinately downregulated in human diabetes. Nat. Genet.

[b25] Morrison A, Hodgetts C, Gossler A, Hrabe de Angelis M, Lewis J (1999). Expression of Delta1 and Serrate1 (Jagged1) in the mouse inner ear. Mech. Develop.

[b26] Nayak G, Goodyear RJ, Legan PK, Noda M, Richardson GP (2011). Evidence for multiple, developmentally regulated isoforms of Ptprq on hair cells of the inner ear. Dev. Neurobiol.

[b27] Odeh H, Hunker KL, Belyantseva IA, Azaiez H, Avenarius MR, Zheng L, Peters LM, Gagnon LH, Hagiwara N, Skynner MJ, Brilliant MH, Allen ND, Riazuddin S, Johnson KR, Raphael Y, Najmabadi H, Friedman TB, Bartles JR, Smith RJ, Kohrman DC (2010). Mutations in Grxcr1 are the basis for inner ear dysfunction in the pirouette mouse. Am. J. Hum. Genet.

[b28] Ricci AJ, Fettiplace R (1998). Calcium permeation of the turtle hair cell mechanotransducer channel and its relation to the composition of endolymph. J. Physiol.

[b29] Riccomagno MM, Martinu L, Mulheisen M, Wu DK, Epstein DJ (2002). Specification of the mammalian cochlea is dependent on Sonic hedgehog. Gene. Dev.

[b30] Sakaguchi H, Tokita J, Naoz M, Bowen-Pope D, Gov NS, Kachar B (2008). Dynamic compartmentalization of protein tyrosine phosphatase receptor Q at the proximal end of stereocilia: implication of myosin VI-based transport. Cell Motil. Cytoskel.

[b31] Salt AN, Inamura N, Thalmann R, Vora A (1989). Calcium gradients in inner ear endolymph. Am. J. Otolaryng.

[b32] Shehu A, Mao J, Gibori GB, Halperin J, Le J, Devi YS, Merrill B, Kiyokawa H, Gibori G (2008). Prolactin receptor-associated protein/17beta-hydroxysteroid dehydrogenase type 7 gene (Hsd17b7) plays a crucial role in embryonic development and fetal survival. Mol. Endocrinol.

[b33] Smyth GK (2004). Linear models and empirical bayes methods for assessing differential expression in microarray experiments. Stat. Appl. Genet. Mo. B.

[b34] Smyth GK, Michaud J, Scott HS (2005). Use of within-array replicate spots for assessing differential expression in microarray experiments. Bioinformatics.

[b35] Stottmann RW, Turbe-Doan A, Tran P, Kratz LE, Moran JL, Kelley RI, Beier DR (2011). Cholesterol metabolism is required for intracellular hedgehog signal transduction in vivo. PLoS Genet.

[b36] Subramanian A, Tamayo P, Mootha VK, Mukherjee S, Ebert BL, Gillette MA, Paulovich A, Pomeroy SL, Golub TR, Lander ES, Mesirov JP (2005). Gene set enrichment analysis: a knowledge-based approach for interpreting genome-wide expression profiles. Proc. Natl. Acad. Sci. USA.

[b37] Team RC (2012). R: A Language and Environment for Statistical Computing.

[b38] Zampini V, Ruttiger L, Johnson SL, Franz C, Furness DN, Waldhaus J, Xiong H, Hackney CM, Holley MC, Offenhauser N, Di Fiore PP, Knipper M, Masetto S, Marcotti W (2011). Eps8 regulates hair bundle length and functional maturation of mammalian auditory hair cells. PLoS Biol.

[b39] Zine A, Van De Water TR, de Ribaupierre F (2000). Notch signaling regulates the pattern of auditory hair cell differentiation in mammals. Development.

